# Evaluation of the Efficacy of CPP-ACP Remineralizing Mousse in MIH White and Yellow Opacities—In Vitro Vickers Microhardness Analysis

**DOI:** 10.3390/dj10100186

**Published:** 2022-10-02

**Authors:** Inês Cardoso-Martins, Sofia Arantes-Oliveira, Ana Coelho, Sofia Pessanha, Paula F. Marques

**Affiliations:** 1 Faculdade de Medicina Dentária, Universidade de Lisboa, Rua Professora Teresa Ambrósio, 1600-277 Lisbon, Portugal; 2 Department of Physics, NOVA School of Science and Technology, Campus Caparica, 2829-516 Caparica, Portugal

**Keywords:** molar incisor hypomineralization, hardness tests, tooth remineralization, tooth hypomineralization, pediatric dentistry, dental enamel, enamel

## Abstract

Remineralization of tooth enamel can be partially achieved by the application of a casein phosphopeptides and amorphous phosphate (CPP-ACP) complex. However, evidence to support its effectiveness in Molar-incisor-hypomineralization (MIH)-affected teeth is scarce. The study’s aim is to evaluate the efficacy of CPP-ACP mousse in remineralizing MIH-affected enamel using a Vickers microhardness test. Two groups of enamel opacities of hypomineralized permanent teeth, white (group A) and yellow (group B) lesions (n = 14), went through a 28-day treatment protocol with GC Tooth Mousse. Before and after treatment, microhardness was measured in three different areas of each tooth (hypomineralized, transition, and outside the hypomineralized area). Data were analyzed using parametric and non-parametric tests with a significance of *p* < 0.05. The mean microhardness values increased in the hypomineralized and transition areas in both groups after the treatment protocol (in group A, 105.38 ± 11.70 to 158.26 ± 37.34; 123.04 ± 22.84 to 156.33 ± 35.70; in group B, 108.63 ± 14.66 to 143.06 ± 22.81; 132.55 ± 20.66 to 146.00 ± 12.88) and the differences pre/post-treatment were statistically significant within each group (*p* < 0.001 for both groups). Between groups, there was a statistically significant difference in the same areas (hypomineralized: *p* = 0.003; transition: *p* = 0.008) with a higher improvement in enamel hardness in group A. Topical application of CPP-ACP showed an increase in the physical strength of the hypomineralized and transition areas of MIH-affected enamel, likely due to an increase in mineral content.

## 1. Introduction

Molar incisor hypomineralization (MIH) is a dental enamel defect of systemic origin that can affect one to four permanent first molars and is frequently associated with affected incisors [1]. Although several likely factors in its development have been investigated, the etiology of MIH remains unclear. It is possible that this pathology is not caused by a specific factor, but rather is a result of several systemic and genetic factors that may act together in an additive or synergistic way [2,3].

Hypomineralization is a qualitative defect that presents enamel-demarcated opacities, which differ greatly in size and shape [3]. They can vary from white to yellow brown, due to an increase in the organic structure on the enamel layer. [4]. Creamy-yellow or whitish-creamy opacities are less porous and variable in depth, while yellow or brownish-yellow defects are of full thickness [5]. Frequently, the high porosity of defects can lead to enamel fracture immediately after tooth eruption as a result of normal occlusal forces [6,7]. A post-eruptive enamel fracture (PEEF) can lead to dentin exposure, the development of dental caries lesions, and excessive chipping and wear of the dentition, requiring teeth restoration [6,7]. Failure of restorative materials due to poor tooth adhesion is likely related to reduced mechanical properties and mineral content observed in teeth with MIH Enhancing the properties of hypomineralized enamel is fundamental to improving the restorative outcomes for these teeth [8,9,10].

There is evidence that the remineralization of tooth enamel can be achieved by the application of a casein phosphopeptide complex and amorphous calcium phosphate (CPP-ACP) [11,12,13,14,15]. The tryptic digestion of milk caseinate produces multiphosphorylated casein phosphopeptides (CPP) that sequester and stabilize calcium phosphate (which is usually highly insoluble) forming a CPP-ACP complex. Due to the higher amount of phosphoseryl residues, this nanocluster of CPP-ACP has a greater calcium-stabilizing capacity than salivary proteins [16]. This molecular complex is readily soluble in saliva, creating a diffusion gradient that allows it to adhere to the tooth surface as well as to the bacteria in the plaque surrounding the tooth. In acidic conditions, through the local release of calcium and phosphate ions, the consistent use of this nanoaggregate allows the maintenance of a supersaturated state in minerals that suppresses demineralization and enhances mineralization [14,17,18].

There is evidence that the application of CPP-ACP pastes decreases the loss of minerals and the depth and width of lesions [19]. Recent studies observed that treating enamel with CCP-ACP prevented the decrease in microhardness after bleaching and that the application of this remineralizing agent reduced surface softening and morphologic changes of the teeth exposed to erosion [20]. Furthermore, it has been shown through Vickers’ microhardness analysis that CPP-ACP favors the remineralization of caries lesions [21]. Although, limited literature exists regarding the remineralizing efficacy of using topical preparations with CPP-ACP in MIH-affected teeth [17,22,23,24]. 

Microhardness tests have great relevance in demineralization and remineralization protocols [25]. To the best of our knowledge, this is the first study that uses the Vickers Microhardness test in the evaluation of MIH-affected teeth after CPP-ACP remineralization protocols. The aim of the present study is to determine, through Vickers microhardness analysis, if our protocol of application of CPP-ACP is effective in remineralizing enamel in different areas of white and yellow MIH opacities. The tested hypothesis was that the in vitro application of CPP-ACP would not increase the microhardness in the transition and hypomineralized areas of white and yellow MIH opacities.

## 2. Materials and Methods

The study sample consisted of 15 severely hypomineralized molar teeth [26], extracted for several reasons such as the presence of severe painful symptoms affecting the patient’s quality of life. After a multidisciplinary approach combining orthodontics, surgery, and pediatric dentistry, the treatment plan revealed that it was favorable to extract the severely hypomineralized first molars. Upon extraction and verbal consent of the guardian of the patient, each tooth was stored and preserved in accordance with ISO conditions (ISO/TS 11405) in a refrigerated 1% chloramine solution for 1 week and then in Hank’s solution BioWhittakerTM. The study protocol was approved by the ethics committee of the Dental School of the University of Lisbon (CE-FMDUL201701).

### 2.1. Sample Preparation

All teeth went through a prophylactic protocol using a polishing brush with pumice paste in a low rotation handpiece (0.04 mg of pumice; 0.8 mL of water) for 60 s, were washed with distilled water for 60 s, and then submitted to an ultrasound bath in ethanol 100% for 60 s using the Bransonic^®^ M2800-E, Emerson, electronic cleaning device. 

**Figure 1:**
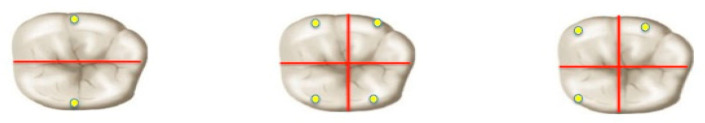
Teeth sections perpendicular to the occlusal surface (in red): 2, 3, or 4 enamel opacities were obtained (in yellow).

Each tooth was encased in an acrylic block using a hot glue pistol and sectioned parallel to the occlusal surface to split the root from the crown using a water-cooled diamond-impregnated circular saw (Isomet, Buehler Ltd., Lake Bluff, IL, USA). Ten teeth were then sectioned perpendicular to the occlusal surface in order to obtain: 2 enamel opacities in 8 teeth; 4 enamel opacities in 1 tooth and 3 enamel opacities in another tooth (Figure 1). The other 5 teeth were not sectioned, and each one presented one enamel opacity.

The 28 opacities from the MIH teeth were allocated in one of the two groups: Group A—14 white hypomineralized opacities; Group B—14 yellow hypomineralized opacities.

Once prepared and while awaiting testing, the samples were stored fully hydrated in Hank’s solution BioWhittakerTM.

**Figure 2:**
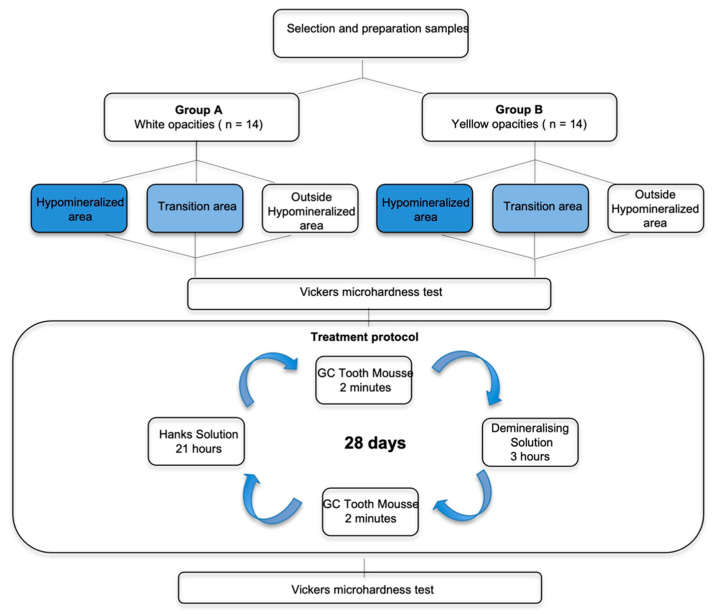
Scheme of the study design.

Figure 2 represents a scheme of the study design.

### 2.2. Vickers Microhardness Test—Pre- and Post-Treatment Analysis

Each sample was removed from Hanks’ solution, washed with distilled water for 5 s, dried with absorbent paper, placed on a holder, and analyzed with the Vickers microhardness test (Vickers Hardness tester, Duramin 5, Struers) before and after treatment. Microhardness was measured in three different areas of each sample (4 measurements were performed in the hypomineralized area, 4 in the transition area, and 4 outside the hypomineralized area), and the results were registered for analysis. 

### 2.3. Treatment Protocol

The treatment procedure was carried out for a period of 28 days [15]. Each of the enamel samples was treated with the remineralizing agent—10% CPP-ACP (GC Tooth mousse TM) for a period of 2 min [15], following which the samples were individually immersed in 2 mL of a demineralizing solution (2.0 mMol/L calcium, 2.0 mMol/L phosphate, 75 mMol/L acetic acid, pH 4.4.) for a period of 3 h [25]. This was followed by the treatment of the samples again with the remineralizing agent for 2 min [15]. Afterward, all the enamel samples were individually immersed in 2 mL of Hank´s balanced salt solution BioWhittakerTM for a period of 21 h. 

The demineralizing agent was replaced every 5 days and the Hanks solution was replaced every 48 h.

### 2.4. Statistical Analysis

Statistical analysis was performed with SPSS v26.0, IBM, New York, NY, USA. The Shapiro–Wilk test for normal distribution evaluation was performed. When data distribution was normal, a paired Student’s *t*-test was performed to evaluate the results (hypomineralized and transition areas in the yellow opacities group; outside the hypomineralized area in the white opacities group). When data were not normally distributed, Wilcoxon Sign Rank was used (outside the hypomineralized area results in the yellow group; hypomineralized and transition areas in the white group). 

Pre and post treatment microhardness differences of the areas in the two groups were analyzed using an independent-samples t-test when the distribution was normal (outside the hypomineralized area) and a Mann–Whitney U test when the data were not normally distributed (transition and hypomineralized areas). A significance level of 0.05 was considered.

## 3. Results

**Figure 3:**
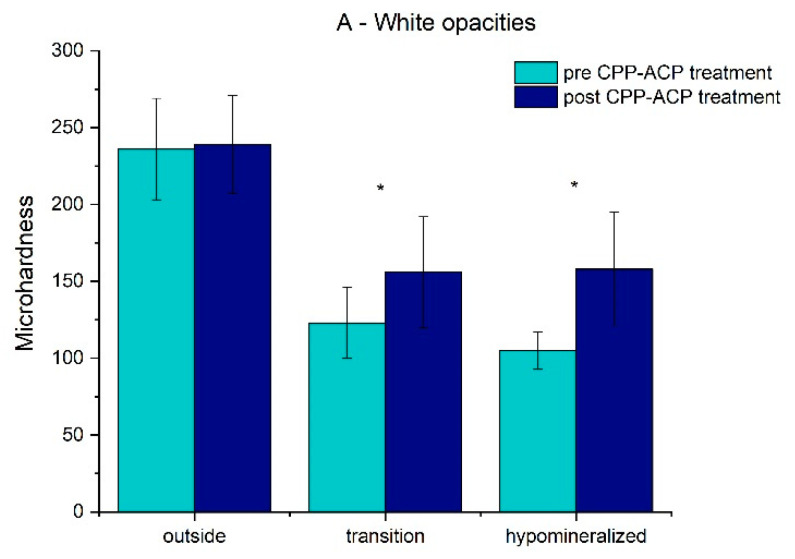
Bar chart of the mean values and standard deviation of microhardness in the white opacities group (* represent statistical significance).

**Figure 4:**
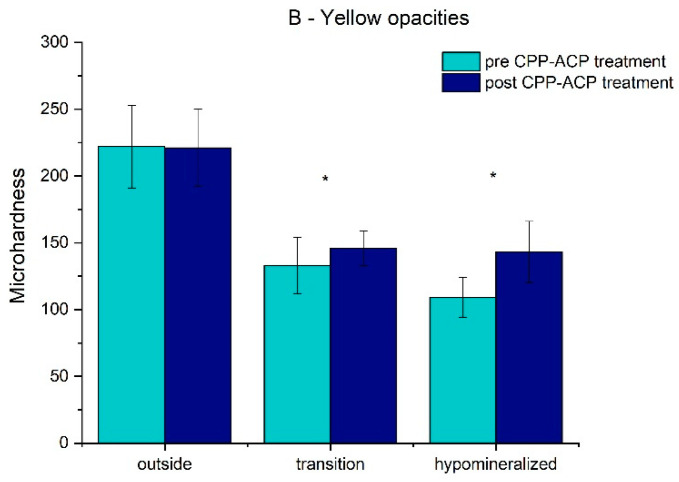
Bar chart of the mean values and standard deviation of microhardness in the yellow opacities group (* represent statistical significance).

The results for the pre/post-treatment microhardness in the different areas of the two groups are presented in Table 1 and Figure 3 and Figure 4.

The mean value and standard deviation of the microhardness for the white opacities teeth (A group) in the hypomineralized and transition areas and outside the hypomineralized area before treatment were 105.38 ± 11.70, 123.04 ± 22.84, and 235.63 ± 32.83, respectively, and after treatment, it was 158.26 ± 37.34, 156.33 ± 35.70, and 238.93 ± 31.71, respectively. For the yellow opacity teeth (B group), before treatment, it was 108.63 ± 14.66, 132.55 ± 20.66, and 221.70 ± 31.28, and after treatment, it was 143.06 ± 22.81, 146.00 ± 12.88, and 220.75 ± 29.14, respectively. For both groups, in the hypomineralized and transition areas, there was a significant increase (*p* < 0.05) in the mean values of microhardness, which suggests an improvement in the enamel mineralization after treatment. There was no statistically significant difference (*p* > 0.05) in the enamel mineralization outside the hypomineralized area for groups A and B before and after treatment.

**Figure 5:**
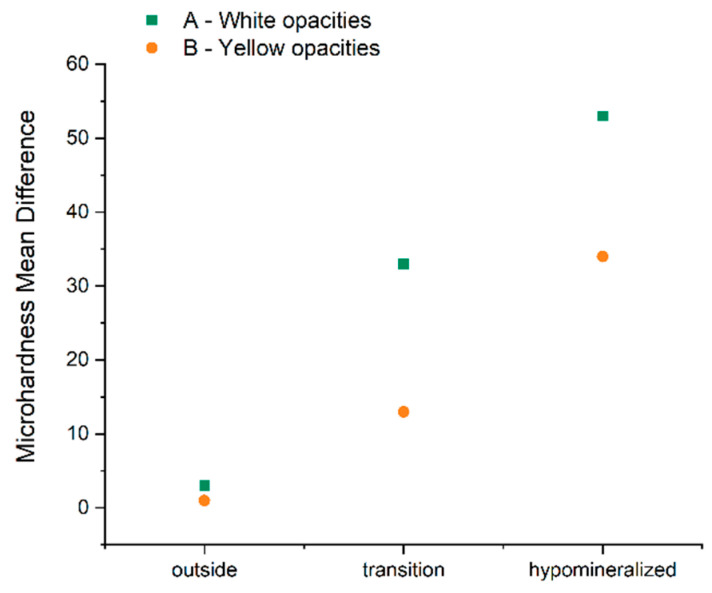
Plot of the mean microhardness differences pre- and post-treatment by region in A—white opacities group—and B—yellow opacities group.

The results for the difference in the pre- and post-treatment microhardness values in the different areas of the two groups are presented in Table 2 and Figure 5.

In group A, the mean and standard deviation of the difference of these microhardness values in the transition and hypomineralized areas and outside the hypomineralized area were 33.28 ± 44.00, 53.00 ± 36.22, and 3.30 ± 26.63, respectively. In group B, they were 13.44 ± 23.97, 34.43 ± 26.79, and 1 ± 24.88, respectively. There were statistically significant differences (*p* < 0.05) in this parameter in the transition and hypomineralized areas between groups. The results were superior in group A, suggesting that there was a higher improvement in the enamel mineralization of the white opacities. There were no statistically significant differences (*p* > 0.05) in this parameter outside the hypomineralized area between groups.

## 4. Discussion

Using Vickers microhardness analysis, we were able to observe that the treatment protocol used for CPP-ACP applications had a positive and significant effect on increasing this mechanical property in the hypomineralized enamel with either white or yellow hypomineralized opacities.

The hypomineralized region of MIH teeth has markedly inferior mechanical properties in comparison to normal enamel. Mahoney et al. [10] showed that the hardness of hypomineralized enamel decreased from the clinically normal cervical enamel to the hypomineralized area occlusally. Microscopically, they observed that the start of the linear decrease in hardness appears to initiate cervically to the visual demarcation of the hypomineralized opacity, which is approximately equivalent to the transition zone. Our study confirms these data.

It also shows that in the hypomineralized and transition areas of the affected teeth, there was a significant increase (*p* < 0.05) in microhardness, which suggests an improvement in the enamel mineralization after CPP-ACP treatment. The microhardness increase in these problematic areas after remineralizing agent application suggests that it is less probable for enamel fracture to occur when MIH teeth are subjected to occlusal loads. The significant improvement in hardness may reduce the difficulty of restoring these teeth and the challenging situation for clinicians concerning the loss of restorative material and tooth tissue [10].

In the present study, the hypomineralized and transition area of MIH teeth showed a significant increase in the microhardness in both the white and yellow opacities groups. The total mineral concentration in teeth with MIH is 5% to 20% lower than that found in healthy molars [9,27,28]. Hypomineralized areas have a significant decrease in P and an increase in C and Mg and exhibit a lower Ca/ C [24]. It has been suggested that there is a positive correlation between the mechanical properties of calcified tissue and its mineral content [10,27]. The incorporation of other extrinsic sources in the apatite structure of enamel crystals results in changes in physico-chemical and mechanical properties of enamel [27].

Angker et al. showed that the mechanical properties of carious dentine are dependent on its mineral content [10,29]. The results of the present study suggest that the improvement of the physical strength of the MIH-affected enamel following CPP-ACP topical application may be due to an increase in mineral content.

The Hanks balanced salt solution, being neutral, emphasizes the effect of CPP, which is a saliva biomimetic but has a remarkable better ability to stabilize calcium phosphate, compared to salivary proteins, through their multiple phosphoseryl residues [13,16,30]. The protocol used for CPP-ACP application was adapted from the protocol used by Shetty et al. in comparative studies on remineralizing agents [15].

A potential limitation of this study was that it was based on an in vitro environment. The effect of salivary enzymes, proteins, pellicle, and dental plaque on demineralization and remineralization cycles in the oral environment were not included. From another perspective, in vitro studies have better control over any confounding variables. 

According to the data obtained, there was more improvement in the enamel microhardness of the affected teeth in the white opacities group compared to the yellow group in the transition and hypomineralized areas. This might suggest that remineralization occurs more superficially in the white lesions group. This difference might be explained by the lower porosity and fewer prismatic cracks of the white lesions when compared to the yellow ones [5,24].

This study highlights the importance of more research using this type of remineralization protocol in MIH-affected teeth. The need for further clinical trials with clearly validated outcome measures must be emphasized prior to the widespread recommendation for the use of CPP-ACP to increase the mineral content of MIH teeth. It would be interesting to conduct future studies on MIH teeth using new remineralizing agents as a biomimetic nano-hydroxyapatite based on the integration of calcium and phosphate at the level of demineralized dental surfaces [31,32].

## 5. Conclusions

Based on this in vitro study’s results, the following conclusions can be drawn:

The analysis of the microhardness using a Vickers test showed a significant increase in the physical strength of the MIH-affected enamel following CPP-ACP topical application, which may be due to an increase in mineral content, thereby rejecting the tested hypothesis.

There was a significant increase in the mineral density of the hypomineralized and transition areas of the enamel after treatment with CPP-ACP tooth mousse in both white and yellow opacities.

There was a superior improvement in the enamel hardness of the MIH-affected teeth in the white opacities group compared to the yellow group in the transition and hypomineralized areas after a 28-day CPP-ACP protocol.

The era of preventive and minimally invasive dentistry dictates the need to develop approaches to remineralize HIM teeth. In future studies, CPP-ACP and other new biomimetic remineralization strategies could be used to improve the structural and mechanical characteristics of MIH-affected teeth.

## Figures and Tables

**Table 1:** Mean microhardness values ± st dev in groups A (white opacities) and B (yellow opacities).

	Outside Opacity			Transition Zone			Hypomineralized Opacity		
	Pre CPP-ACP	Post CPP-ACP	*p*-value	Pre CPP-ACP	Post CPP-ACP	*p*-value	Pre CPP-ACP	Post CPP-ACP	*p*-value
**Group A**	235.63 ± 32.83	238.93 ± 31.71	0.358	123.04 ± 22.84	156.33 ± 35.70	**<0.001**	105.38 ± 11.70	158.26 ± 37.34	**<0.001**
**Group B**	221.70 ± 31.28	220.75 ± 29.14	0.997	132.55 ± 20.66	146.00 ± 12.88	**<0.001**	108.63 ± 14.66	143.06 ± 22.81	**<0.001**

**Table 2:** Mean microhardness differences in group A (white opacities) and B (yellow opacities).

Microhardness Differences Mean Values ± St Dev	Outside Opacity		Transition Zone		Hypomineralized Opacity	
**A group**	3.30 ± 26.63	*p* = 0.385	33.28 ± 44.00	***p* = 0.008**	53.00 ± 36.22	***p* = 0.003**
**B group**	1 ± 24.88		13.44 ± 23.97		34.43 ± 26.79	

## Data Availability

Not applicable.
